# Analyzing postpartum patients’ attitudes towards medication for weight management: a mixed-methods analysis

**DOI:** 10.3389/frph.2026.1968399

**Published:** 2026-10-21

**Authors:** Julia Erickson, Levi J. Anderson, Laine Boitos, Bryan Aaron, Samantha Schon, Ashley Bartell

**Affiliations:** 1 Division of Maternal Fetal Medicine, Department of Obstetrics and Gynecology, University of Michigan, Ann Arbor, MI, United States; 2 Department of Obstetrics and Gynecology, University of Michigan, Ann Arbor, MI, United States; 3 Division of Reproductive Endocrinology and Infertility, Department of Obstetrics and Gynecology, University of Michigan, Ann Arbor, MI, United States

**Keywords:** cardiometabolic health, GLP-1, postpartum, pregnancy, weight

## Abstract

**Introduction:**

Pregnancy and the postpartum period present unique stressors that can be barriers to weight loss efforts. The landscape of weight management is rapidly evolving, but without specific understanding or consideration of postpartum people's needs and attitudes towards newer pharmaceutical options (e.g., GLP-1 agonists). We aim to characterize postpartum people's perspectives on weight management interventions in the weeks to months following birth. This study assesses the attitudes of postpartum people with a BMI >30 kg/m2 towards weight management in the immediate postpartum period.

**Methods:**

This was a prospective, observational study leveraging mixed-methods techniques to extract the opinions and stances of postpartum people from a single institution with a BMI >30 kg/m2 via semi-structured interviews. A total of three focus groups and two one-on-one interviews were conducted. Thematic qualitative analysis was performed to identify emergent themes, followed by a sentiment analysis to quantitatively compare the emotive lexical context of each major theme.

**Results:**

We qualitatively identified four major themes during the interviews: attitudes about medication (“Medication”), attitudes towards and experiences with weight fluctuation (“Weight”), tools for losing weight (“Tools”), and concerns about long-term health (“Concern”). Quantitatively, participant statements about “Tools” were the most positive, with polarity scores (0.33 ± 0.27), while sentences related to “Concern” were the most negative (polarity scores 0.02 ± 0.25). Terms representing the emotions “joy,” “anticipation,” and “trust” were notably overrepresented compared to other emotions in sentences categorized as discussing “Tools.” These positive stances were reaffirmed in our qualitative review, though participants were skeptical regarding the implications of weight loss medications on breastfeeding. They also conveyed concern about short-term use of medications, favoring a sustainable multimodal approach. Overall, participants expressed overwhelmingly positive attitudes towards weight management interventions in the postpartum period, specifically in conjunction with other lifestyle adjustments and support from medical and familial outlets.

**Discussion:**

In semi-structured interviews, postpartum participants with a BMI >30 kg/m2 favored a comprehensive plan for weight loss and management—including pharmacologic interventions and social supports or lifestyle alterations—starting in the weeks to months postpartum. Though they highlighted lingering concerns regarding safety and efficacy of pharmacologic interventions in this context, participants were open to integrating medications as one of many tools to optimize long-term cardiometabolic well-being.

## 1 Introduction

Approximately one in four pregnant patients in the U.S. enter pregnancy with a BMI ≥30 kg/m^2^ (1, 2), which is positively correlated with cardiometabolic disease risk. Patients are aware of weight retention's impact on their long-term health; higher BMI is associated with greater dissatisfaction with postpartum body weight and composition (3). Weight retention is perceived as a persistent problem that affects physical health, self-image, and future pregnancy risks (4).

Despite awareness of the health risks, many patients report difficulty accessing or adhering to structured weight management programs (5). The Society for Maternal-Fetal Medicine recommends that women be encouraged to reach their pre-pregnancy weight by 6–12 months postpartum (6). Structured lifestyle interventions—including behavioral counseling, digital health programs, and combined diet and physical activity strategies—are effective for reducing postpartum weight retention, improving body composition, and lowering cardiometabolic risk, though results are more variable outside of trial settings (7). Highly effective solutions such as newer weight loss medications and bariatric surgeries can achieve 6%–30% total body weight loss in non-pregnant, non-lactating adults (8). Weight loss surgeries are not recommended for patients who are immediately postpartum due to ongoing physical transitions. Weight loss medications such as the new GLP-1 and GLP-1/GIP agonist classes are hypothetically available, but have yet to be studied in this specific population. Preliminary lactation data is reassuring and prescription rates are increasing for these newer medications, contributing to the need for a better understanding of patients’ overall perspective on pharmaceutical interventions as well as other options and supports for postpartum weight management (9–11).

Postpartum patients with elevated BMI commonly express strong interest in losing weight after pregnancy (12). However, attitudes towards intervention in the first six weeks postpartum are poorly characterized. As new medications are increasingly utilized for weight loss, studies evaluating postpartum patients’ feelings about them in the context of their weight loss journey are limited. We sought to better understand postpartum patients’ perspectives on weight management.

## 2 Materials and methods

We conducted a prospective, observational study leveraging mixed-methods techniques in a cohort of patients receiving postpartum care at a single institution. Using a semi-structured interview format, we conducted three 45–60 min focus groups (*n* = 4, 4, 5, respectively) and two one-on-one interviews. All patients presenting for a postpartum visit were screened by a research team member to determine eligibility (live-birth, BMI ≥30 kg/m^2^, English-speaking). A study team member approached eligible patients during their clinic visit to explain the goal of the focus groups. If the patient expressed interest, verbal consent was obtained and a short survey was administered to obtain further demographic characteristics and pregnancy history. The University of Michigan Institutional Review Board deemed this study exempt.

A semi-structured interview guide developed by the research team asked open-ended questions about postpartum weight and general attitudes towards weight loss medications (Appendix 1). Focus groups were conducted on Zoom by a trained study team member with extensive experience in motivational interviewing and focus group conduction. Two participants completed one-on-one interviews with the facilitator on Zoom using the same interview guide due to limitations in scheduling and availability. Conversations were recorded and transcripts were cleaned, de-identified, and manually coded for themes.

Inductive qualitative analysis was completed by two coders trained by content experts in qualitative research methods via formal university-level training and mentorship from senior physician-scientists. Coders assigned emergent themes to each turn-at-talk with at least one content word to saturation independently before moving to consensus discussions and formal codebook definitions (see Appendix 2 for glossary of linguistic terminology) (13, 14). They did this using intentionally decontextualized turns-at-talk to limit co-dependent inferences specific to a given speaker. To address any discrepancies, the senior author considered the input and reasoning from each coder and determined the most appropriate final categorization. Where multiple codes were applicable for the same text, the senior author determined which was most appropriate via discussion with the coding team. Only a single code was ultimately applied via adjudication. With respect to positionality, one coder was present for all interviews where camera presence was requested but not required. The second coder, however, had no interaction with participants. Both coders have extensive experience working in women's health and obstetric research that encompasses the full spectrum of peri-reproductive aged participants.

A sentiment analysis was performed to compare the emotive lexical context of each major theme, providing an objective complement to the qualitative analysis. Participant talk was tokenized into sentences and then into words. At the sentence level, polarity was calculated using the Sentimentr package within the R statistical computing environment (The R Foundation, Vienna, Austria). Quantified polarity was used to define negative versus positive polarity sentences. An ANOVA of polarity values by theme was performed to test for differences in the mean polarity across combinations of each of the four themes. A *post-hoc* Tukey Honestly Significant Difference Test was performed for individual comparisons. The positive and negative polarity sentences were then separately dissociated into words. Stop and filler words were filtered using the Stringr package. Remaining content words within each polarity grouping were then associated with emotions based on categories represented in the National Research Council (NRC) lexicon, which operationalizes Plutchik's wheel of emotions (15). Descriptive statistics were performed on emotion categories by qualitative themes. A Chi-squared test of the distribution of emotions was also conducted, with *post-hoc* testing of individual emotion-theme pairings. A Bonferroni correction was applied for multiple comparisons. The measures reported (themes, NRC categories, and polarity scores) were considered to be summative and independent from intra-speaker variance, as supported by use of highly tokenized data. Inferential analyses were used in accordance with other work utilizing corpus-based techniques that decontextualize words and phrases. However, given the small sample size of participants, these analyses should be viewed as exploratory. All quantitative analyses were done in RStudio (Posit, PBC, Boston, MA).

## 3 Results

Of 66 patients screened for eligibility, 44 met eligibility criteria, 27 of whom were approached about participating. Approaches were limited by staff availability and clinic acuity on given days. Of 20 patients who agreed to participate, 15 attended and completed a focus group or one-on-one interview. The mean age of participants was 33.7 ± 4.8 years. The mean parity was 3.1 ± 1.8. The mean BMI was 40.9 ± 8.6 kg/m^2^. Complete demographic information is in Table 1. All participants (*N* = 15, 100%) had given birth less than three months prior to being approached to participate. Participants’ pregnancy complications and chronic conditions are shown in Table 2.

**Table 1:** Patient-reported demographic information (*N* = 15).

Characteristic	n (%) or Mean ± SD
Age, years	33.67 ± 4.81
Gravida	3.47 ± 1.96
Parity	3.07 ± 1.83
BMI, kg/m^2^	40.86 ± 8.57
Race	
American Indian or Alaskan Native	0 (0)
Asian	1 (7)
Black or African American	5 (33)
Hispanic/Latino	0 (0)
Middle Eastern or North African	1 (7)
Native Hawaiian or Pacific Islander	0 (0)
White	8 (53)
Education Level	
High school/GED	3 (20)
Bachelor's degree	3 (20)
Master's degree	3 (20)
Trade school	1 (7)
Associate's degree	2 (13)
PhD	1 (7)
Some college	1 (7)
Other	1 (7)
Marital Status	
Single	4 (27)
Married	10 (67)
Divorced	1 (7)
Employment Status	
Full-time	9 (60)
Part-time	1 (7)
Contract/Temporary	1 (7)
Unemployed	2 (13)
Self-employed	1 (7)
Prefer not to say	1 (7)
Annual Household Income	
Less than $25,000	0 (0)
$25,000-$50,000	4 (27)
$50,000-$100,000	3 (20)
$100,000-$200,000	6 (40)
More than $200,000	1 (7)
Prefer not to say	1 (7)
Number of Dependents	
None	1 (7)
1	7 (47)
2–3	2 (13)
4 or more	5 (33)

**Table 2:** Patient-reported pregnancy information (*N* = 15).

Variable	n (%)
Length of Time Since Giving Birth	
<3 months	15 (100)
Mode of Delivery	
Vaginal	7 (47)
First-time cesarean	4 (27)
Repeat cesarean	4 (27)
Pregnancy Complications**	
Fetal growth restriction	1 (7)
High blood pressure in pregnancy	7 (47)
Gestational diabetes	3 (20)
Preterm birth (<37 weeks)	4 (27)
None	3 (20)
Other	1 (7)
Chronic Conditions**	
Chronic hypertension	5 (33)
Pre-existing diabetes	2 (13)
Asthma	1 (7)
Anxiety	3 (20)
Depression	2 (13)
Autoimmune disease	0 (0)
Cardiac disease	1 (7)
Opioid use disorder or other substance use disorder	0 (0)
None	5 (33)

** Pregnancy complications and chronic conditions are not mutually exclusive. Participants were asked to “select all that apply” when self-reporting.

Qualitative analysis identified four larger categories of themes present across all transcripts (Table 3): attitudes about weight loss medications (“Medication”), attitudes towards/experiences with weight fluctuation (“Weight”), tools for losing weight (“Tools”), and concerns about long-term health (“Concern”). There were 117 total coded focus group participant turns: 50 (42.7%) “Medication”-coded, 30 (25.6%) “Weight”-coded, 24 (20.5%) “Tools”-coded, and 13 “Concern”-coded (11.1%). Participants produced 136 sentences in “Medications” turns, 46 in “Weight” turns, 43 in “Tools” turns, and 44 in “Concerns” turns. Sentences about “Tools” had the highest polarity scores (0.33 ± 0.27), followed by “Weight” (0.12 ± 0.31) and “Medication” (0.10 ± 0.35), while “Concern” had the lowest scores (0.02 ± 0.25). An ANOVA showed a statistically significant difference in mean polarity scores across themes (F = 32.4, *P* < .01). Pairwise comparison of polarity by theme further demonstrated that all inter-theme differences were significant to at least *P* = .01 except the comparison between “Weight” & “Medication” (*P* = .77, Table 4).

**Table 3:** Major themes and sub-themes from qualitative coding.

Theme	Sub-Theme
Attitudes about medication	Medication as a tool for weight management Medication and the potential impacts on newborn Medication related to body image Medication versus bariatric surgery Societal perceptions of weight loss medications
Attitudes towards/experiences with weight fluctuation	Societal perceptions Weight and mental health Weight when pregnant versus when not pregnant
Tools for losing weight	Lifestyle interventions Medications Religion/culture Social supports
Concerns about long-term health	Motivation to be healthy for oneself Motivation to be healthy for newborn Weight related to future family planning

**Table 4:** ANOVA comparisons.

Comparison	Mean difference in polarity score	95% Confidence Interval	*P* value
Medication:Concern	0.08	0.01–0.15	.01
Tools:Concern	0.31	0.23–0.40	<.01
Weight:Concern	0.10	0.03–0.18	<.01
Tools:Medication	0.23	0.16–0.30	<.01
Weight:Medication	0.02	−0.04–0.08	.77
Weight:Tools	−0.21	−0.29–−0.13	<.01

It is important to differentiate between “medication” as a sub-theme within the major “Tools” category compared to the broader “Attitudes about medication” major theme. Qualitatively, the “Tools” category was utilized when participants were discussing objective information about medications for weight management. Conversely, the more commonly utilized “Attitudes towards medication” code was used when participants shared their personal thoughts on pharmacologic strategies for weight management.

The word distribution in positive polarity environments by theme and NRC-matched emotion are shown in Figures 1,2. Positive terms form the majority of words spoken by participants in the “Tools”-coded turns, while positive and negative terms are relatively balanced in the other themes (Figure 1). Joy, anticipation, and trust are notably overrepresented compared to the other emotions in “Tools”-coded turns (Figure 2). Anger is the least common emotive association overall, but is relatively overrepresented in “Medication”-coded turns. Fear, disgust, and sadness are similarly distributed across “Concern,” “Weight,” and “Medication”-coded talk within positive polarity environments. This collectively demonstrates a relatively positive stance towards “Tools,” both at the sentence level (based on polarity scores) and the word level (based on the emotive association). A Chi-squared test of NRC term distribution by theme was significant (X^2 52.1, *P* < .01), with the relative prevalence of word-level positivity in “Tools”-coded talk as the only significant difference on *post-hoc* testing (Residual 3.40, adjusted *P* = .02). This relationship is visualized by both sentential polarity and lexical valence in Figure 3.

**Figure 1:**
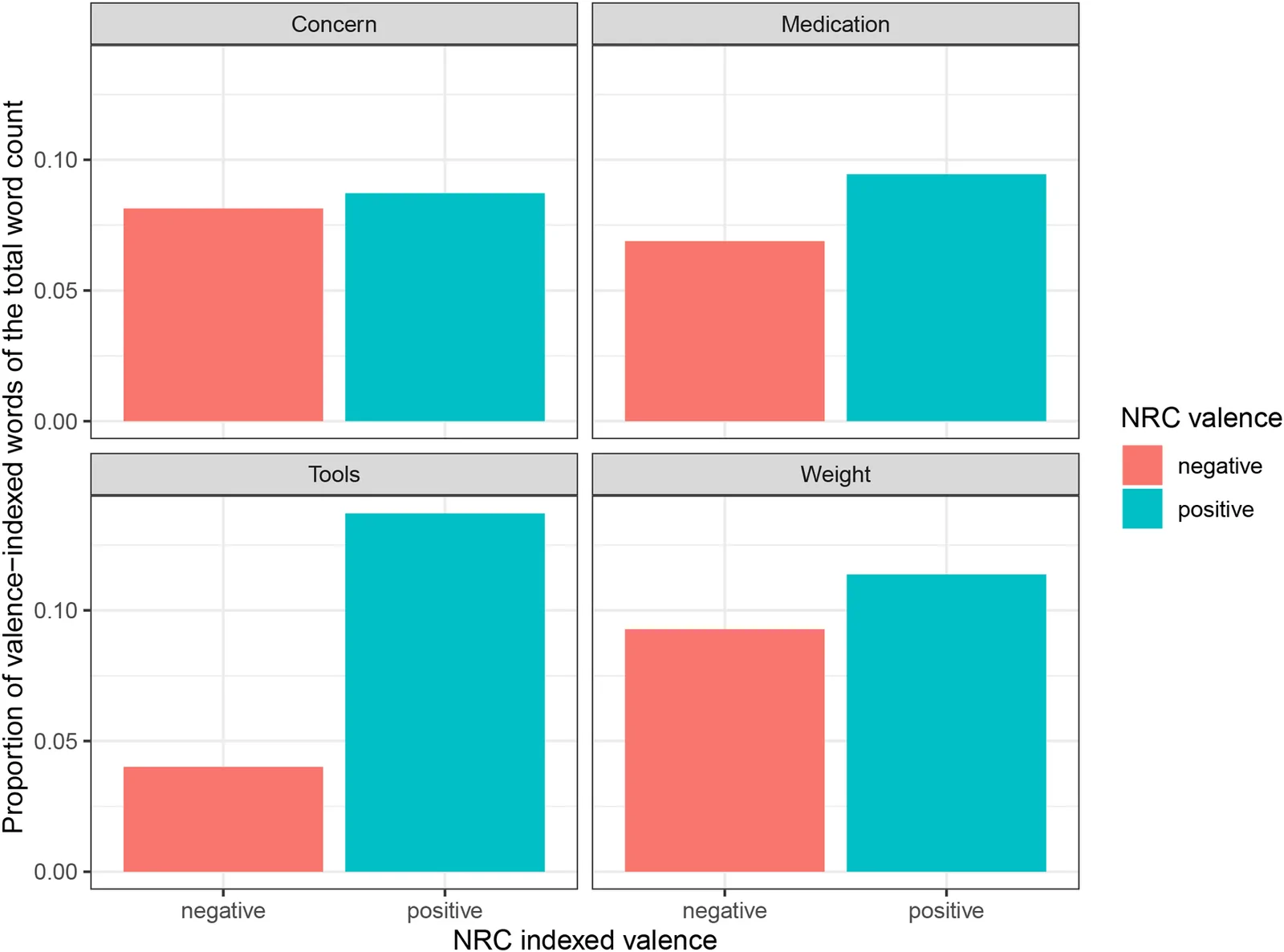
Proportion of positive and negative valence-indexed words by four main themes identified in qualitative analysis. The generic positivity and negativity assignments (i.e., valence) of the words are given by theme. Positivity and negativity are defined by the NRC (National Research Council) lexicons. The numerators for the proportions represent the number of transcribed words matching a word in the lexicon for a given valence, where the denominator is the total number of participant-produced, transcribed words. These lexicons (positivity and negativity) are mutually exclusive, so no terms were counted twice. The four panels are the major themes identified in emergent thematic analysis.

**Figure 2:**
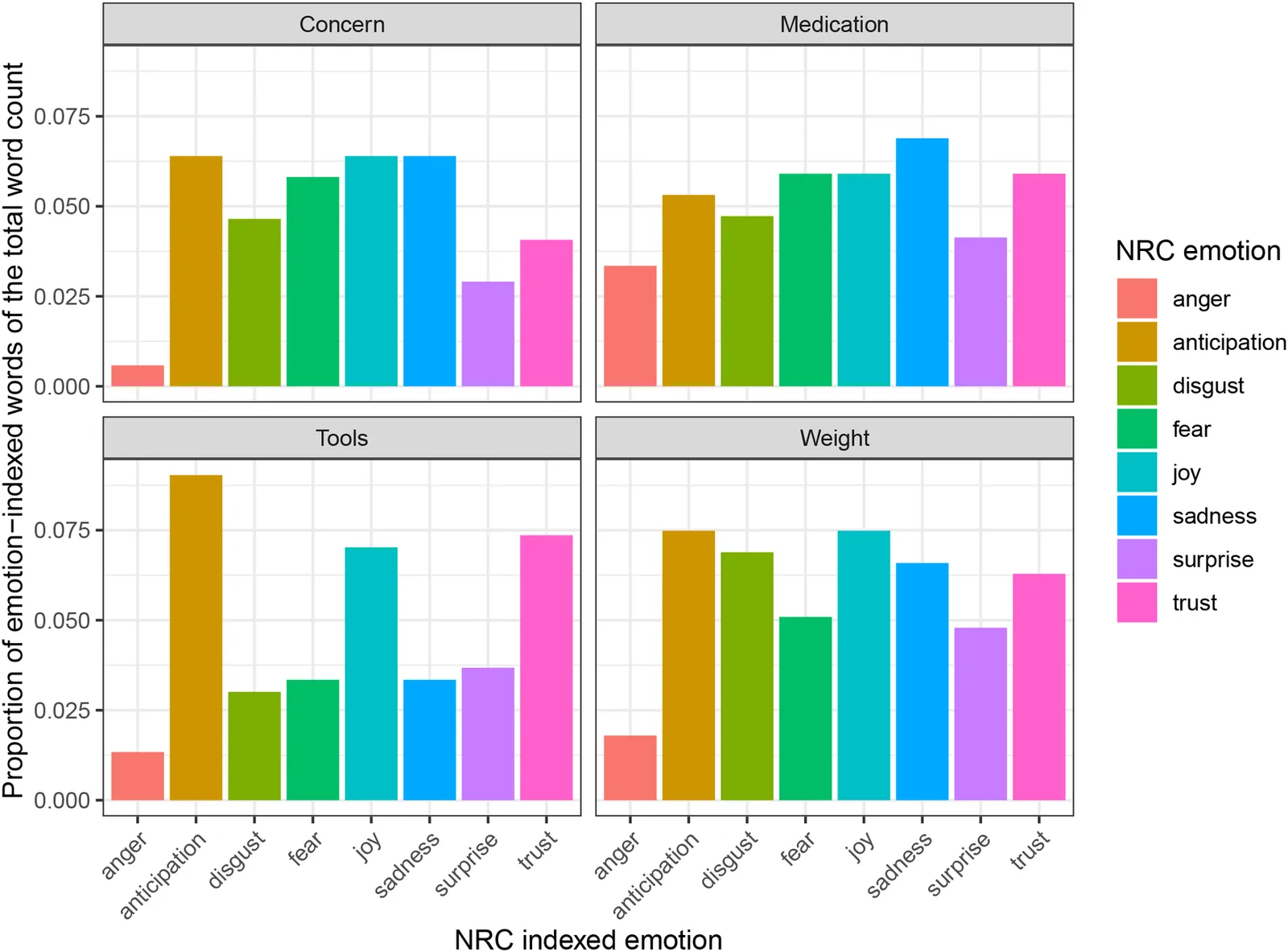
Proportion of NRC emotions by four main themes identified in qualitative analysis. Emotive terms are defined by the NRC (National Research Council) lexicons. The numerators for the proportions represent the number of transcribed words matching a word in the sub-lexicon for a given emotion, where the denominator is the total number of participant-produced, transcribed words. Transcribed words could be counted in multiple categories if a word was part of multiple sub-lexicons. The four panels are the major themes identified in emergent thematic analysis.

**Figure 3:**
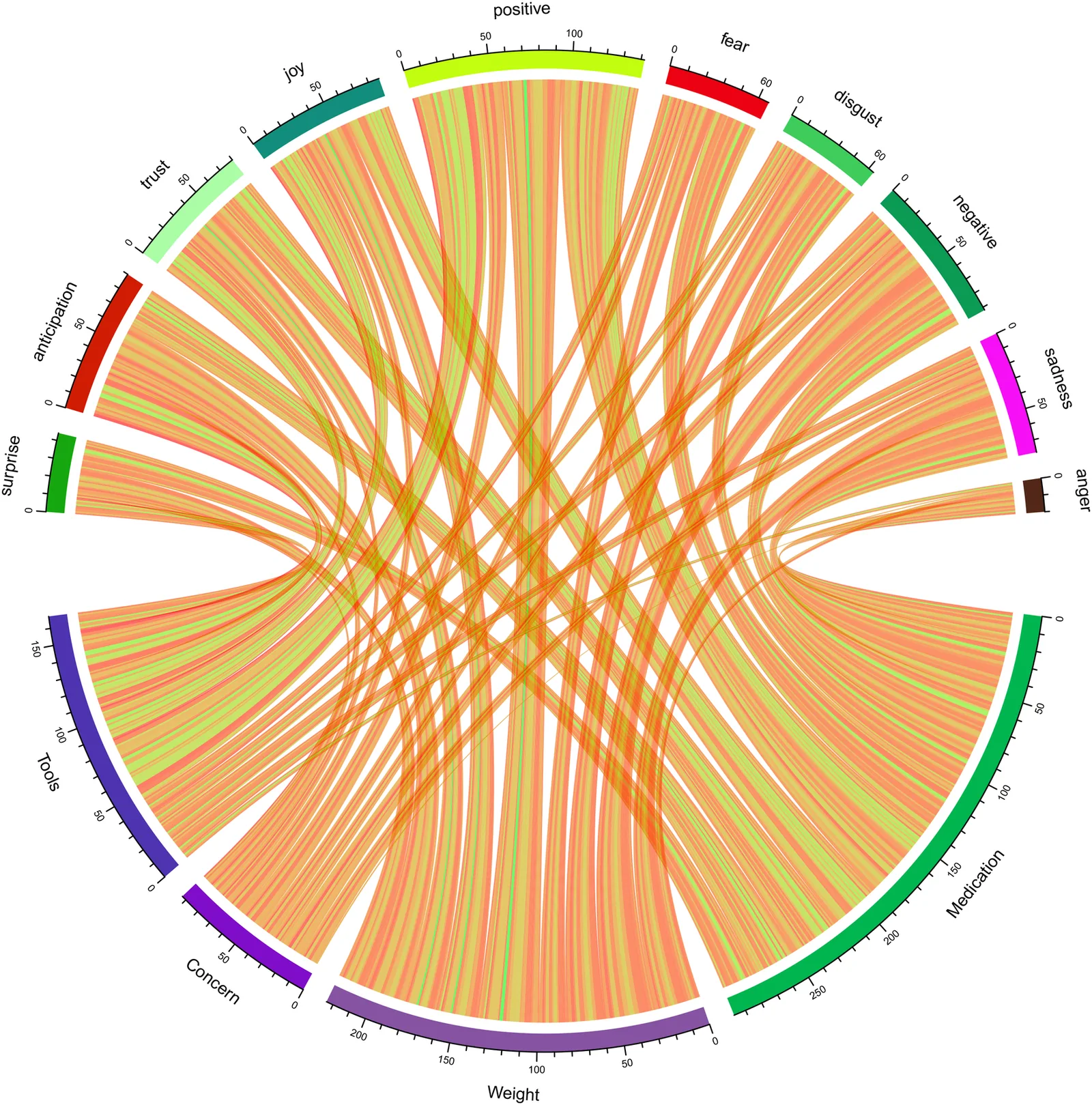
Associations between qualitative themes and emotive terms. This chord diagrm represents the relationships between emotions (Top colored bands), major themes from our thematic analysis (Bottom colored bands), and positive/ negative sentiment. Emotions are defined by the NRC (National Research Council) lexicons. The four major themes were identified by our emergent thematic analysis of focus group transcripts. Themes and emotions are connected by bands that demonstrate if the words used by participants in focus groups occurred in an overall positive (green) or negative (red) sentiment environments. For example, a majority of green in the band connecting an emotion (e.g., joy) and a theme (e.g., tools) suggests that an intersection of that emotion, theme, and valence (e.g., words that were categorized as “joy” in talk about “tools” tended to have positive contexts). Numbers represent the total number of words in each emotion or thematic category.

### 3.1 “Medication”

Participants were queried on their attitudes regarding the use of pharmaceutical treatments for obesity and weight management, among other topics related to weight loss and postpartum support. While GLP-1 receptor agonists were not explicitly mentioned by the facilitator, participants independently drew associations to a number of currently available name-brand medications. A near majority of natural, contentful turns-at-talk were participants’ stances on medications. Discussions included medication as a tool for weight management, the potential impacts on a newborn, body image, medication versus bariatric surgery, and societal perceptions.

Participants were generally positive about pharmacological means in the postpartum period to facilitate a return to pre-pregnancy weight, seeing it as one of many ways to move towards their goal weight; i.e., one of many tools in a toolkit as opposed to a single solution.

“All it was was a nudge in the right direction”—Participant O

Those with a negative stance towards medication used preferred methods like diet and exercise. They expressed skepticism about the long-term sustainability of medication-assisted weight loss, stating that although it may take longer, the ultimate results may be more promising.

“I don't think [the weight loss medications] are worth it. I would just say go natural and try. It might take longer time, but it helps, like, overall health. Is how I see it. Not just, like, losing weight, but it's gonna help you in the long term, too.”—Participant M

Some participants viewed the short-term benefits of weight management medication as not worth the potentially disappointing long-term results after discontinuing medication.

### 3.2 “Weight”

All groups began by asking participants to reflect on their most pressing postpartum health concerns. Without prompting, a large proportion of participants cited their feelings about weight fluctuation. The facilitator further probed participants to think about how this fluctuation relates to the greater picture of their postpartum health. Responses included societal perceptions of weight gain, mental health, and weight when pregnant versus not.

Regarding weight gain relative to pregnancy versus outside of pregnancy, many participants cited feeling a loss of control. Despite the excitement of being pregnant, it was difficult to look past the weight gain regardless of the reasons for it.

“Seeing that scale continue to rise, although I was carrying a human, yes, I still was like, oh my gosh. And then, especially getting to the numbers that I was never at. Made me feel..I don't know. It made me just feel like at that moment, defeated, I will say. A little.”—Participant N

Conversely, some noted it was easier to grapple with physical changes when they could attribute them to growing a baby. In these scenarios, the external locus of control allowed patients to view weight gain as a sign of progress.

“Oh my gosh, I'm, like, proud of it, like, I have a reason. You know, for me to have the weight on now, versus…you know, any other time. This, you can look at the baby and say, okay, this is the reason why, but any other time, not the case.”—Participant H

These participants noted feelings of “pride” or strength related to weight in these moments following birth.

### 3.3 “Tools”

Participants were asked to discuss their existing strategies for postpartum weight management. Many shared experiences with prior weight loss attempts outside of pregnancy, as well as efforts to manage weight during inter-pregnancy intervals. Most commonly, participants shared intentional lifestyle shifts; however, challenges arose when a strategy was no longer applicable in pregnancy.

“..[N]ow I have to worry about if I drop my calories, like, I can't go any lower, because then I'm breastfeeding, so I almost feel like, I guess I'll just deal with this later, but then, like, how long do you put it off? And then, like I said, I got pregnant again…”—Participant L

Separately, many participants equated weight loss goals with overall postpartum wellness, and agreed that a solid support system was vital to success for both. Regarding past successes, Participant B shared “you gotta have a support system.” Whether to help with motivation or accountability, all agreed that having trusted people to encourage the continuous prioritization of health and well-being was instrumental to postpartum recovery.

### 3.4 “Concern”

Embedded into all aspects of these focus groups was a general sentiment that participants wanted to be mindful of their long-term health related to weight and other factors. While not explicitly asked about it, many participants freely cited a general sense of the “bigger picture” related to their postpartum journey and weight management desires. Specifically, general attitudes towards future family planning and the role of weight in conception were prevalent.

“I'm worried about my weight, because I want to have more babies… I don't want problems next pregnancy”—Participant M

Separately, many participants connected their weight to their ability to be present and care for their children. Many acknowledged a duality to their overall drive to “be healthy”; some focused more heavily on health in relation to personal self-goals, while others attributed their motivation to the desire to be there for their newborn. There were polarizing thoughts on this topic, with one participant sharing “you feel guilty if you try to lose any weight, because then you feel like, in what ways am I harming my baby?” (Participant D), while others equated weight with longevity, noting that they are willing to do whatever is necessary to guarantee they will live a long, healthy life with their child.

“I need to be able to be on top of my game. I'm not willing to, you know, not take care of myself so I don't see my children graduate. You know, I have to be able to do those things, and part of that is taking care of myself and getting rid of some of this extra fluffiness I've been carrying around my entire life.”—Participant C

Additional participant quotes are in Supplementary File 1.

## 4 Discussion

Through a mixed-methods approach, we found that participants had an overwhelmingly positive attitude towards tools for weight management, including the use of medication, when integrated into a comprehensively supportive environment. More specifically, asking about medication in isolation as a strategy for weight management yielded mixed emotions in both qualitative and quantitative analyses. Long-term maternal and neonatal health were expressed priorities, with emphasis on sustainable lifestyle change, compatibility with breastfeeding, and anticipating future pregnancies.

Previous studies have demonstrated that postpartum people face a number of barriers related to weight management following delivery, including postpartum depression and other mental health disorders, lack of familial and social support, low health literacy, childcare needs, and time constraints (16, 17). Our analysis found that patients valued multimodal, behavior-based, and socially informed approaches to weight loss, with medications complementing these strategies.

Existing research shows that the sensitivity of the postpartum period and its associated transitions present obstacles for women attempting to lose weight and manage their health (18). While women commonly alter many of their practices during pregnancy to focus on their own well-being, priorities shift to the health of their baby following delivery (18). Importantly, women are interested and willing to put effort towards such personal lifestyle shifts, but the barriers experienced with a newborn present multiple challenges (19). Therefore, programs embedded into existing maternal and child health services delivered through a variety of formats with a flexible approach were the most favored by this population (19). This trend was prevalent among our study's participants, with many taking pride in their pre- and intra-pregnancy lifestyle priorities, but finding it difficult to maintain these practices following delivery.

The stigma surrounding weight, especially in relation to pregnancy and postpartum, is rampant in today's healthcare landscape (20). While health behavior change is often an integral part of care for people entering pregnancy with a higher body weight (21), this need not come at the expense of relationships with their healthcare team. Patients who experience stigma or discrimination from a provider are more likely to report negative feelings towards their care, ultimately impacting the quality of their care and potentially leading them to engage in unhealthy and dangerous behaviors (20). The complexity of this issue does not stop at the healthcare level. Social support in a home environment plays an integral role in the sustainability of healthy behavior changes, increasing the likelihood of long-term wellness (22, 23). Women have historically noted that simply feeling “seen” by others contributes to monumental shifts in feelings of confidence and capability (24).

Furthermore, the complexity of the postpartum period mustn't go unaddressed. Weight loss is one part of the larger picture of postpartum health, which is simultaneously impacted by broader health-related behavioral changes and outcomes such as changing nutritional needs, physical demand, cardiometabolic indicators, sleep, and psychological well-being. In future research, we acknowledge the importance of incorporating robust screening for disordered eating, body-image outcomes, psychological well-being, and weight-stigma-sensitive care to provide the highest standard, comprehensive care to this population.

Considering this context, we hypothesize that the most pragmatic approach to weight management combines lifestyle shifts, social support, and pharmacological interventions (e.g., GLP-1 agonists). Numerous focus group participants shared their prior experiences with weight loss medications, citing past success being hindered by the nature of the pregnancy and postpartum periods. Additionally, they noted improved results when combining lifestyle changes with pharmacologic interventions. Other participants who had never used medication for weight loss were more inclined to view it as a successful tool when used in conjunction with other techniques, especially ongoing education and support (25).

Our findings suggest that women are eager to find tools to help manage weight, especially in the context of pregnancy and postpartum. GLP-1s are becoming an increasingly popular treatment option for numerous health conditions, including weight management, diabetes, and cardiovascular disease. As the pharmaceutical landscape continues to evolve, significant effort should be made to incorporate the lived experiences of postpartum women to ensure this oft-neglected population gains and maintains access to important therapeutically beneficial options for weight management. Moreover, clinical and public health interventions designed to target postpartum weight intervention should reinforce principles of community engagement, social support, and self-efficacy.

Our mixed-methods approach allowed us to reaffirm qualitative findings quantitatively. Quantitative analysis showed an overwhelmingly positive valence associated with speech referencing tools that could be used for weight management. The emotion most highly related to this category was anticipation, followed by trust and joy. In other words, not only do people feel positive about finding tools for weight management, they also desire them, trust their ability to do what they’re supposed to, and experience some feelings of joy about them. Importantly, the positive attitude towards “Tools” for weight should not be attributed to GLP-1 medications alone. This code encompassed a number of strategies for weight management, namely lifestyle shifts, social, and cultural supports. Rather than interpreting this finding as a positive attitude towards medication alone, these findings support the greater conclusion that participants expressed particularly positive language towards a multimodal approach to weight management that incorporates various resources and supports. Furthermore, the strong results from this small group justify replicating these methods in a larger, multi-site setting using other forms of transcribed speech (e.g., message boards and social media) to diversify the results, thus broadening generalizability and more intentionally incorporating women's voices into the co-design of postpartum lifestyle interventions.

### 4.1 Strengths and limitations

Strengths of this study include the variety of patients’ obstetric experiences and outcomes. Additionally, the intimate nature of our small focus groups allowed participants to more fully and completely participate in discussions. Finally, through complex linguistic analysis, our quantitative findings reaffirmed the attitudes and ideas that participants shared in a qualitative setting, demonstrating that our mixed-methods approach allows for a more comprehensive representation of this population's sentiments regarding attitudes towards weight loss.

Importantly, this was a small study sampling patients from one medical institution, with some demographic categories represented by only one participant; therefore, the generalizability of our findings are limited to this group. We used coding techniques and analytic tools that treated the data as a corpus in an attempt to limit the effect of intra-speaker co-dependencies. However, units of talk cannot be assumed to be fully speaker-independent and the number of speakers should still be considered in contextualizing these findings. Though our inferential analyses were intended to capture the decontextualized approach to words and phrases used, they should be validated on larger participant sets. Additionally, this study was limited by lack of discussion of postpartum eating disorders and behaviors. Given the known psychological and physiological sensitivity of this time period, it is important to consider these factors in future work in the field. Overall, we feel that this preliminary data demonstrates a strong need for additional research on postpartum patients’ weight management perspectives and provides a framework for future studies.

### 4.2 Conclusions

While many strategies exist for postpartum weight management, our study found that a comprehensive plan—including combined pharmacologic interventions, social supports, and lifestyle alterations—yielded the most positive responses from patients, whereas attitudes towards medications alone were heterogeneous. Though the safety and efficacy of such pharmacologic interventions in the postpartum period are under-studied, our findings suggest there is a definite need for resources and additional research in this area.

## Data Availability

The original contributions presented in the study are included in the article/Supplementary Material, further inquiries can be directed to the corresponding author.
