# Supplementary material for: Analyzing postpartum patients’ attitudes towards medication for weight management: a mixed-methods analysis
Source: Front Reprod Health. 2026 Oct 21;8:1968399. doi: 10.3389/frph.2026.1968399 (PMC13731105; doi:10.3389/frph.2026.1968399)
Supplement: Supplementary file 1 [file Supplementaryfile1.docx]

**Appendix 1:** **Interview guide for focus groups**

**Topic: Postpartum Weight**

**Primary questions:**

- What were your biggest concerns about your health postpartum?
- Were you worried about weight gain during pregnancy? Why or why not?
- Did you try to lose weight postpartum? Why or why not?
- How would you describe the relationship between weight gain in and around pregnancy and your health?
- How is weight gain in and around pregnancy different than in other times in life?

***Further prompts:***

- What influenced these concerns?
- Was there anything that was particularly helpful or useful in these efforts?

**Topic: General Attitudes Towards Weight Loss Medications**

**Questions:**

- What are some words and phrases you associate with people who use weight loss medications?
- How do the new weight loss medications affect body image?
- How do weight loss medications compare to weight loss surgery? Would you consider one over the other? Why?
- Are the new weight loss medications a good or bad thing for our society?
