# Supplementary material for: Analyzing postpartum patients’ attitudes towards medication for weight management: a mixed-methods analysis
Source: Front Reprod Health. 2026 Oct 21;8:1968399. doi: 10.3389/frph.2026.1968399 (PMC13731105; doi:10.3389/frph.2026.1968399)
Supplement: Supplementary file 2 [file Supplementaryfile2.docx]

**Quotes from focus groups by domain**

**Attitudes About Medication**

**Medication as a tool (+):** “I think what would be really appealing, to me is the fact that there might be something that might be able to help me lose weight a little bit faster, so that I could feel a little bit more like myself, build my confidence back up, especially after having a baby with a new… another new baby, you know, another new baby, another new body that you're trying to get used to, and I feel like this would be something that would be good to kind of, you know. Help build confidence back up. And… and kind of be a kickstart to something for better results down the line, you know, for natural working out and stuff, so I think it would be really good in that… in that aspect.” - *Participant F*

**Medication as a tool (+):** “if that one injection makes me lose all my weight? Perfect. Magic. Perfect.” - *Participant G*

**Medication as a tool (+):** “I think they're hopeful, that's one word, so I think it's hopeful, I think, just even the…[people taking weight loss medications] having the ability to…not give up, like, that's… That's literally what it's showing. It's like, okay, obviously, I've tried this and this, this is not working, let me…let me just keep going, let me keep trying. So I think that's just, that's showing…Willingness to, like, just…Just being courageous, honestly.” - *Participant N*

**Medication as a tool (+):** “somebody just said to me yesterday, "well, she definitely didn't take medication to lose weight". As if it was a negative thing. And to me, I'm like, this is [crosstalk] But I, you know, I'm like, this is a woman who's weighing her options, and who needs help, and I think that's the big thing. I think that there can be a lot of people who take medications out of ease. Maybe it's not necessarily a necessity, but they take it out at ease, and it just makes the job easier, which I can't even dog on, but as I mentioned before, I'm always moving, and I have always been overweight, and my diet has always been a topic of conversation that here I am, Class 3 obesity. I need help. Okay? And that's what I think when I think of people who are taking medic- weight loss medication, I'm like, they need help. And that is what medication is for” - *Participant O*

**Medication as a tool (+):** “All it was was a nudge in the right direction” - *Participant O*

**Medication as a tool (–):** “I don't think, they're worth the weight loss medications are worth. I would just say go natural and try. It might take longer time, but it helps, like, overall health. Is how I see it. Not just, like, losing weight, but it's gonna help you in the long term, too.” – *Participant M*

**Medication as a tool (–):** “I'm very skeptical. I'm very hesitant. I make jokes all the time that we're all gonna end up with cancer in 15 years, because nobody knew that these drugs could give us cancer” - *Participant J*

**Medication related to body image (+):** “I think it's good, because, I mean, what better way to, feel better about yourself if you, you know, if you're out of this depression because of the weight gain?” - *Participant D*

**Medication related to body image (+):** “Oh, man, I tell you what, when I was taking some weight loss medication, what, maybe like a year and a half ago? A year ago? I felt like Beyonce.” - *Participant O*

**Medication related to body image (+):** “And you want to know what's crazy? I was, I was still insanely overweight. I was still, I was right on the cusp between Class 3 obesity and Class 2 obesity, and I remember seeing a picture of myself, and I was extremely overweight, and I was shocked that that's how I looked, because I felt so good. And I didn't even let it tear me down, I was like, okay, I get it, like, I still have, you know, 80 pounds I need to lose, like, I understand. But I remember being shocked, because I was like, I look so damn good. Every single day, I look incredible. And then I see a picture of myself, and I was like, that's what I look like? That does not translate. So, I think it has a massively positive impact on body image.” - *Participant O*

**Medication related to body image (+):** “if medication can make me feel better, losing weight, feel confident, be hot again. So, yeah, I like it.” - *Participant G*

**Societal perceptions (+):** “I know this is gonna sound a little silly, but Serena Williams has been talking about using weight loss medication. That caused a profound, like, shift in my own mindset, because obviously she was, like. Playing professional tennis and working out, like, training, like, 5 hours a day, and still needed That support, and seeing it as, like, one tool in a bigger toolkit. is something that gives me a more positive association, because I think my negative association is you're just doing this one thing, but you're not including these other aspects of long-term health. A professional athlete who utilizes it, helps me see it as something which is, simply one thing that we're able to do in order to help ourselves, not the only thing.” - *Participant K*

**Societal perceptions (–):** “it's, like, the cheating form, you know, of…of dieting or whatever. Oh, you're just gonna take a shot, you're not actually gonna, you know, do anything” - *Participant C*

**Societal perceptions (–):** “people assume it's the easy way out” - *Participant L*

**Medication vs Surgery (+)**: “I think that I would be opposed to more of surgery, just because of the risk associated with it” - *Participant D*

**Medication vs Surgery (+)**: “I can say I can try the medication before I do surgery, because you never know if…How you will come out” - *Participant B*

**Medication vs Surgery (+)**: “I've had surgery before, but now that I have kids, it, like, scares me a lot more.” - *Participant I*

**Medication vs Surgery (+)**: “Surgery feels more extreme. It feels like, risky and dangerous in different types of ways, and there's no, like, going back.” - *Participant K*

**Medication vs Surgery (+)**: “you could stop the medications whenever you want to, but the surgery, not necessarily. It's indefinite, it's definite, yeah. Like, once you sign and sign the paper and it's done, it's done. There's no, okay, I can't stop it. Like, you've already… went through with it.” - *Participant N*

**Attitudes Towards/Experiences with Weight Fluctuation**

**Weight in pregnancy vs not (+):** “looking at my baby and knowing why I gained the weight I did” - Participant A

**Weight in pregnancy vs not (+):** “Oh my gosh, I'm, like, proud of it, like, I have a reason. You know, for me to have the weight on now, versus…you know, any other time. This, you can look at the baby and say, okay, this is the reason why, but any other time, not the case.” - *Participant H*

**Weight in pregnancy vs not (+):** “I feel like even with your postpartum, you don't want to be too hard on yourself, at least for me, personally, I'm trying not to be. And just giving myself time to, like, heal before focusing on, you know, your weight, and just being that subconscious about, like, oh my gosh, you know, I still have the baby fat” - *Participant N*

**Weight in pregnancy vs not (–):** “I was 20 pounds away from being able to go skydiving and found out I was pregnant, and now I'm 80 pounds away from being able to go skydiving, like, come on.” - *Participant C*

**Weight in pregnancy vs not (–):** “I was just really worried that people were gonna be, like, shaming about putting on weight, so I was, like, very, very probably overly closely watching how much I put on, to a degree that was, like, worrisome to me. Just because people are more, like, thinking about and talking to you about your weight more frequently.” - *Participant K*

**Weight in pregnancy vs not (–):** “seeing that scale continue to rise, although I was carrying a human, yes, I still was like, oh my gosh. And then, especially getting to the numbers that I was never at. Made me feel...I don't know. It made me just feel like at that moment, defeated, I will say. A little.” – *Participant N*

**Weight in pregnancy vs not (–):** “I would say I'm more discouraged about my weight in pregnancy, where now I'm feeling much more hopeful… I felt way more hopeful in a sense that I felt like I could bounce back” - *Participant O*

**Societal perceptions (–):** “this whole world is morbidly obese, but we're still gonna make clothes for Barbies.” - *Participant C*

**Societal perceptions (–):** “Just sometimes you compare yourself to other person. Like, you cannot… cannot deny that. When you see, like, other women just give birth, and she's, like, so fit!” - *Participant G*

**Societal perceptions (–):** “It almost kind of makes you feel like you have to be in that specific mold. Instead of actually embracing your own journey. It makes you feel like you're not moving at the speed of everybody else.” - *Participant F*

**Tools for Losing Weight**

**Religion/Culture (+)**: “culturally, like, after you have baby, there's so many different types of foods and stuff that like, my mom wants me to eat, and it's hard to track those” - *Participant M*

**Social Support (+)**: “And you gotta have a support system.” - *Participant B*

**Social Support (+)**: “a lot of the encouragement comes from my husband, honestly, because he's a big walker, and he's a big, you know, and so, that's actually something that I guess helps me, or is or is helpful? Is that, like, I have this other person Kind of encouraging me, and, like, Almost reminding me sometimes” - *Participant J*

**Lifestyle Interventions (+):** “I would just track, and I would drop pretty, pretty quickly. And then now I have to worry about if I drop my calories, like, I can't go any lower, because then I'm breastfeeding, so I almost feel like, I guess I'll just deal with this later, but then, like, how long do you put it off? And then, like I said, I got pregnant again…” - *Participant L*

**Concerns About Long-Term Health**

**Motivation to be healthy for their baby (+)**: “it was managing the weight that I had gained and also trying to make sure I was producing enough milk. So that has been kind of a hard thing to balance between the two of trying to manage how not to keep putting on the weight, but also, be able to produce enough milk for my baby.” - *Participant D*

**Motivation to be healthy for their baby (+):** “you feel guilty if you try to lose any weight, because then you feel like, in what ways am I harming my baby?” - *Participant D*

**Motivation to be healthy for their baby (+):** “I'm already looking at gonna be 58 when he graduates…you know, I need to be able to be on top of my game. I'm not willing to, you know, not take care of myself so I don't see my children graduate. You know, I have to be able to do those things, and part of that is taking care of myself and getting rid of some of this extra fluffiness I've been carrying around my entire life.” - *Participant C*

**Weight related to conception (+):** “I'm worried about my weight, because I want to have more babies… I don’t want problems next pregnancy” - *Participant M*

**Weight related to conception (+):** “we have to do IVF for us to have kids, so typically, like, they want you below, like, a certain, like, BMI threshold, just because, you know, like, the heavier you are, the more risk to you and to potential baby. And we would also like more kids, so…” - *Participant I*

(+)=positive attitude; (–)=negative attitude
