# Supplementary material for: Analyzing postpartum patients’ attitudes towards medication for weight management: a mixed-methods analysis
Source: Front Reprod Health. 2026 Oct 21;8:1968399. doi: 10.3389/frph.2026.1968399 (PMC13731105; doi:10.3389/frph.2026.1968399)
Supplement: Supplementary file 3 [file Supplementaryfile3.docx]

| **Term** | **Examples and/or Definition** |
| --- | --- |
| Conversational turn | Contiguous talk produced by a single speaker consisting of sentence, phrase, and word-level units |
| Content words | Words with an identifiable denotation semantically independent from their conversational context, excluding discourse markers or disfluent utterances |
| Discourse markers only or disfluent utterances | Words without contextually independent meaning that serve interactional purposes such as marking affiliation or indicating sustained attention (e.g., “Uh huh,” “Umm,” “Yep”) |
| Tokenized talk | Talk that has been decontextualized and separated into minimal units at the level of the word or phrase |
| Quantified polarity | Presence versus absence of negation terms expressed as a sum of negation terms |
| Negation terms | Words or phrases that reverse the polarity of a phrase (e.g., “No,” “Not,” “Never”) |
| Negative polarity phrases, sentences, or turns | Communicative units with some degree of negation, instantiated by negation terms |
| Positive polarity phrases, sentences, or turns | Communicative units without identifiable negation terms |
| Polarity (term, not analytical method) | A quantification of positivity vs negativity for a given unit of talk; a high polarity score suggests relative absence of negation, where the presence of negation terms results in a more negative score |

**Appendix 2: Glossary of Linguistic Terminology**
